# CaFÉ: A Sensitive, Low-Cost Filtration Method for Detecting Polioviruses and Other Enteroviruses in Residual Waters

**DOI:** 10.3389/fenvs.2022.914387

**Published:** 2022-07-04

**Authors:** Hanen Belgasmi, Stacey Jeffries Miles, Leanna Sayyad, Kimberly Wong, Chelsea Harrington, Nancy Gerloff, Angela D Coulliette-Salmond, Ratigorn Guntapong, Ratana Tacharoenmuang, Apiradee Isarangkul Na Ayutthaya, Lea Necitas G. Apostol, Ma.Anne-Lesley D. Valencia, Cara C. Burns, Gloria-Rey Benito, Everardo Vega

**Affiliations:** 1 Polio and Picornavirus Laboratory Branch, Division of Viral Diseases, Centers for Disease Control and Prevention, Atlanta, GA, United States; 2 Cherokee Nation Assurance, Tulsa, OK, United States; 3 U.S Public Health Service, Rockville, MD, United States; 4 Department of Medical Science, Enteric Viruses Section, National Institute of Health, Nonthaburi, Thailand; 5 Research Institute for Tropical Medicine, Muntinlupa City, Philippines; 6 Pan American Health Organization, World Health Organization, Washington, DC, United States

**Keywords:** poliovirus, environmental surveillance, filtration, two-phase separation, vaccine-derived poliovirus, enterovirus, wastewater

## Abstract

Acute flaccid paralysis (AFP) surveillance has been used to identify polio cases and target vaccination campaigns since the inception of the Global Poliovirus Eradication Initiative (GPEI) in 1988. To date, only Afghanistan and Pakistan have failed to interrupt wild poliovirus transmission. Circulation of vaccine-derived polioviruses (VDPV) continues to be a problem in high-risk areas of the Eastern Mediterranean, African, and Southeast Asian regions. Environmental surveillance (ES) is an important adjunct to AFP surveillance, helping to identify circulating polioviruses in problematic areas. Stools from AFP cases and contacts (>200,000 specimens/year) and ES samples (>642 sites) are referred to 146 laboratories in the Global Polio Laboratory Network (GPLN) for testing. Although most World Health Organization supported laboratories use the two-phase separation method due to its simplicity and effectiveness, alternative simple, widely available, and cost-effective methods are needed. The CAFÉ (Concentration and Filtration Elution) method was developed from existing filtration methods to handle any type of sewage or residual waters. At $10–20 US per sample for consumable materials, CAFÉ is cost effective, and all equipment and reagents are readily available from markets and suppliers globally. The report describes the results from a parallel study of CAFÉ method with the standard two-phase separation method. The study was performed with samples collected from five countries (Guatemala, Haïti, Thailand, Papua New Guinea, and the Philippines), run in three laboratories–(United States, Thailand and in the Philippines) to account for regional and sample-to-sample variability. Samples from each site were divided into two 500 ml aliquots and processed by both methods, with no other additional concentration or manipulation. The results of 338 parallel-tested samples show that the CAFÉ method is more sensitive than the two-phase separation method for detection of non-polio enteroviruses (*p*-value < 0.0001) and performed as well as the two-phase separation method for polioviruses detection with no significant difference (*p*-value > 0.05). The CAFÉ method is a robust, sensitive, and cost-effective method for isolating enteroviruses from residual waters.

## 1 INTRODUCTION

Since the launch of the GPEI in 1988, the number of annual polio cases has decreased by >99% (Hovi et al., 2012; Bigouette at al., 2021). Afghanistan and Pakistan are the only two countries with reservoirs of endemic wild poliovirus (WPV) (Fomban leke et al., 2020). In 2019, the World Health Organization (WHO) developed the Polio Endgame Strategy 2019–2023 (Organization, 2019), with the goal to end transmission of wild poliovirus type 1 (WPV1), stop circulation of vaccine-derived poliovirus (cVDPV) outbreaks, strengthen immunization and health systems, certify eradication of WPV, and contain all polioviruses, as the major priorities. In 2021, GPEI developed the Polio Eradication Strategy 2022–2026 (Organization, 2021), which describes a comprehensive set of actions that will position the GPEI to deliver on the goals from 2019—2023 polio endgame strategy. Specifically, the goals are to interrupt poliovirus transmission in endemic countries, stop cVDPV transmission, and prevent outbreaks in non-endemic countries. In 2021, endemic WPV1 transmission was restricted to parts of Pakistan and Afghanistan; there were two cases in Pakistan and three in Afghanistan. WPV1 related to a Pakistan lineage was detected in Malawi AFP case with onset in November 2021 (Organization, 2022). The reported cVDPV cases in 2020 and 2021 were 1,113 and 628, respectively, from 33 countries, indicating that the cVDPV cases have exceeded the number of WPV globally (Organization, 2019). VDPV outbreaks were reported in many countries in the African Region, as well as the Philippines, Sichuan province of China and the Middle East (Greene et al., 2019; Alleman et al., 2020; Alleman et al., 2021b).

Acute flaccid paralysis (AFP) plays an important role in the GPEI by providing clinical surveillance for suspected polio cases. Poliovirus infection is confirmed by analysis of stool specimens (Asghar et al., 2014) but is dependent on having a sensitive AFP surveillance system. Environmental surveillance (ES) can identify polio circulation where AFP surveillance is absent or unreliable (Organization, 2019). ES has played a key role in documenting the elimination of WPV from Egypt, India (El Bassioni et al., 2003; Organization, 2015a) and Nigeria (Kalkowska et al., 2020). The collection and processing of samples from sewage or wastewater have been used for many years to supplement AFP surveillance in many countries (Organization, 1988).

ES continues to play an important role in the eradication of WPV from the remaining polio-endemic countries of Pakistan and Afghanistan by identifying residual WPV transmission. In Israel, an ES system has been used since 1988 as an early warning system to detect imported WPV (El Bassioni et al., 2003; Organization, 2003; Blake et al., 2018). Similarly, ES can also assist in the detection of VDPVs; such strains arise from multiple genetic changes and sustained transmission of polioviruses derived from OPV, especially in areas with suboptimal vaccine coverage (Organization, 2015a). The ES method currently recommended by the WHO GPLN involves the processing of 500 ml sewage by polyethylene glycol (PEG)/dextran two-phase separation method (Pöyry et al., 1988; Hovi et al., 2001; Hovi et al., 2005; Esteves-Jaramillo et al., 2014; Ndiaye et al., 2014; Organization, 2015b). A main strength of this method is that it is relatively simple and does not require complex equipment. However, dextran is manufactured by only two companies. The Bag Mediated Filtration System (BMFS) was designed to enable sampling and field processing of large water volumes (Zhou et al., 2018; Coulliette-Salmond et al., 2019; Estívariz et al., 2019; Fagnant-Sperati, et al., 2020), but the BMFS method requires equipment that is not commercially available.

The objective of this study was to compare two environmental surveillance sample concentration and processing methods for poliovirus detection: the standard two-phase separation and a filtration method (e.g., Concentration and Filtration Elution method: CaFÉ) that was developed based on a previous filtration method (Iwai et al., 2006; Nakamura et al., 2015). The CaFÉ method was developed and optimized at the CDC (Atlanta, United States) using samples collected from Haïti and Guatemala (parallel testing). Subsequently, it was compared to the two-phase separation method in pilot studies in the Philippines, Thailand and using samples collected from Papua New Guinea (pilot testing).

## 2 MATERIALS AND METHODS

### 2.1 Parallel and Pilot Studies Site Selection

#### 2.1.1 Parallel Study

**FIGURE 1 |:**
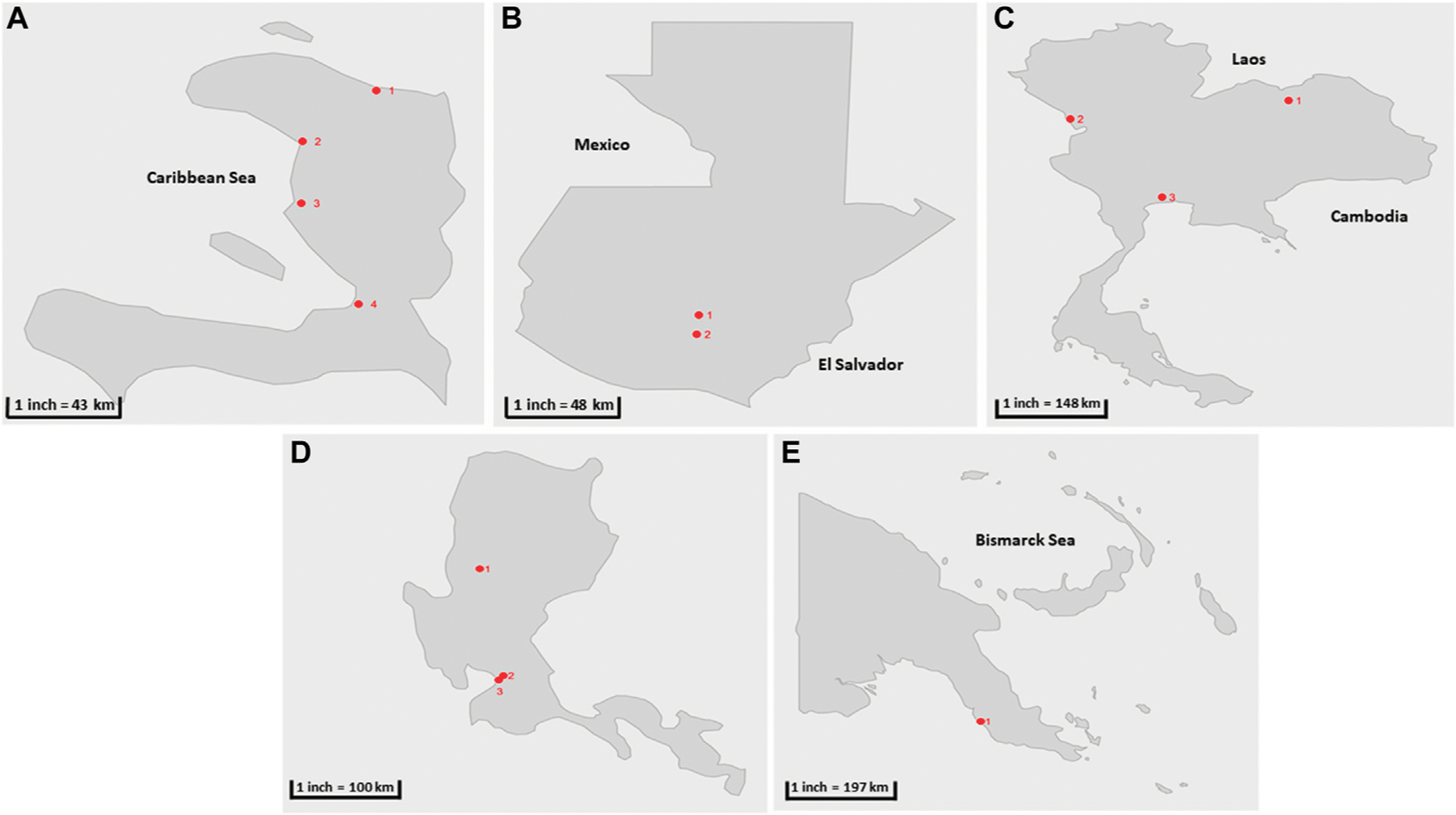
Geographical location of sample collection cities during the parallel and pilot studies (shown in red circles) **(A)** Haïti 1) Cap-Haïtien. 2) Gonaïves, 3) Saint Marc, 4) Port au prince **(B)** Guatemala: 1) San Juan Sacatepéquez, 2) Villa Nueva **(C)** Thailand 1) Udonthani, 2) Tak, 3) Bangkok. **(D)** The Philippines: 1) Baguio, 2) Manila, 3) Quezon City. **(E)** Papua New Guinea: 1) Port Moresby.

Parallel testing between the two-phase separation and CaFÉ methods was conducted at CDC-Atlanta, using samples collected from Haïti between December 2017—December 2019 (*n* = 144), twelve sampling sites in four coastal cities in Haïti—Port au Prince, Gonaïves, Saint Marc, and Cap-Haïtien were selected (Alleman et al., 2021a) (Table 1; Figure 1A), and samples from Guatemala from November 2018—August 2019 (*n* = 53). Seven sampling sites were selected in two major cities in Guatemala—San Juan Sacatepéquez, a municipality located in the northwest of Guatemala City. Villa Nueva located south of Guatemala City (Table 1; Figure 1B). These two cities were selected for evaluation, according to the GPEI environmental surveillance guidelines (Organization, 2003), based on 1) population, 2) and road accessibility during the rainy and dry seasons.

Geographical coordinates for each site were measured by a Montana 600, a handheld GPS device (Garmin International, Olathe, KS, Unites States), and watershed populations were estimated using the Worldpop spatial demographic dataset (Department of Geography and Environment, University of Southampton, United Kingdom) (Novel-t, 2021).

#### 2.1.2 Pilot Study

Six sites were selected in Thailand from previously established sites for poliovirus surveillance (Table 1; Figure 1C) with sample collection conducted between February 2019-July 2019 (*n* = 58) and processing at the National Institute of Health Laboratory (NIH). Two sites were selected in the country`s capital Bangkok: the Din Daeng site (in the inner zone of Bangkok) and the Nong Khaem site (in the western zone of Bangkok). Two sites were selected from the Udon Thani province: Nongsim and Huay Mak Khaeng and two sites were selected in Mae Sot district, in Tak province at the border with Myanmar. This district is known for the presence of a large hospital and is a transportation hub between the two countries.

Three collection sites were included for the pilot study at the Research Institute for Tropical Medicine Laboratory (RITM) in the Philippines, two in Metro Manila: one in Quezon City and the other one located in the city of Manila. The third collection site is in Baguio City. Samples were collected from October 2018—December 2020 (*n* = 54) (Table 1; Figure 1D).

Three collection sites were selected in Papua New Guinea: in Port Moresby, Gerehu Sewage Lagoon (GSL), Waigani Sewage Lagoon (WSL) and Joyce Bay Treatment Plant (JTP). The samples were shipped to RITM for processing and included in pilot testing collected from October 2018—December 2019 (*n* = 29) (Table 1; Figure 1E).

### 2.2 Sample Concentration

#### 2.2.1 Sample Collection and Frequency

Two 1-L wastewater samples were collected approximately once every 4 weeks at each sampling site in Haïti, Guatemala, Thailand, Philippines, and Papua New Guinea, using the GPLN/WHO recommended sampling method, the grab method with a swing sampler (NASCO, Fort Atkinson, WI) with 1-L Nalgene bottles (Organization, 2015c). In the grab method, the sample was collected at one point in time, also it is a more quantitative method that allows estimation of the system`s detection sensitivity (Organization, 2003). Time, date of collection, and sample temperature as well as weather conditions on collection day and previous day were recorded. All samples were stored at 2–8°C immediately after collection and transported to the laboratory or stored at ≤ −20°C until shipment. All specimens were shipped frozen on dry ice and stored at ≤ −20°C until processing.

#### 2.2.2 Pre-analytical Processing

Upon arrival at the laboratory, sample temperature was recorded using a temperature gun (Etekcity, Anaheim, CA, United States), and duplicate 1-L environmental water samples for each collection site and collection month were thawed at room temperature (25°C) for 24 h before processing, combined, and mixed for 15 min. Water quality was analyzed using 30 ml aliquot of each sample, by measuring the pH, total dissolved solids and salinity. Two aliquots of 500 ml were measured for processing with two-phase separation and CaFÉ methods, remaining sample volume was stored at −20°C for additional testing if needed.

#### 2.2.3 Two-phase Separation Method

The samples were concentrated using the two-phase separation method as described previously (Organization, 2015a). Briefly, the sample was centrifuged for 20 min at 6,500 x *g* at 4°C. The supernatant is added to a beaker and pH was adjusted to 7.0–7.5. The resulting pellet, if any, is stored at 4°C for later addition to the concentrate. Two polymers were added to the clarified sample: polyethylene glycol (6,000) (29% w/v; Sigma-Aldrich, St. Louis, MO) and dextran T40 (22% w/v; Pharmacosmos, Holbaek, Denmark), as well as 5M sodium chloride (Sigma-Aldrich), and then mixed for 1 hour at 10,000 rpm at 4°C. the obtained homogenous mixture is added to a glass separatory funnel and left to stand overnight at 4°C, to allow the polymers separation and form two distinct phases. After incubation, the lower phase and interphase were collected into a 50 ml centrifuge tube (approximately 10–15 ml). The stored pellet from the initial centrifugation was suspended in the concentrate, which is then treated with chloroform (20% v/v; Sigma-Aldrich), agitated for 20 min at 10,000 rpm at room temperature (25°C), then centrifuged for 20 min at 1,500 x *g* at 4°C. Antibiotics (100 IU/ml penicillin and 100 μg/ml streptomycin and 50 μg/ml gentamycin) were added to the isolated supernatant. The concentrate was inoculated into cell culture for poliovirus isolation the same day, as described below.

#### 2.2.4 Filtration Method (CaFÉ)

**FIGURE 2 |:**
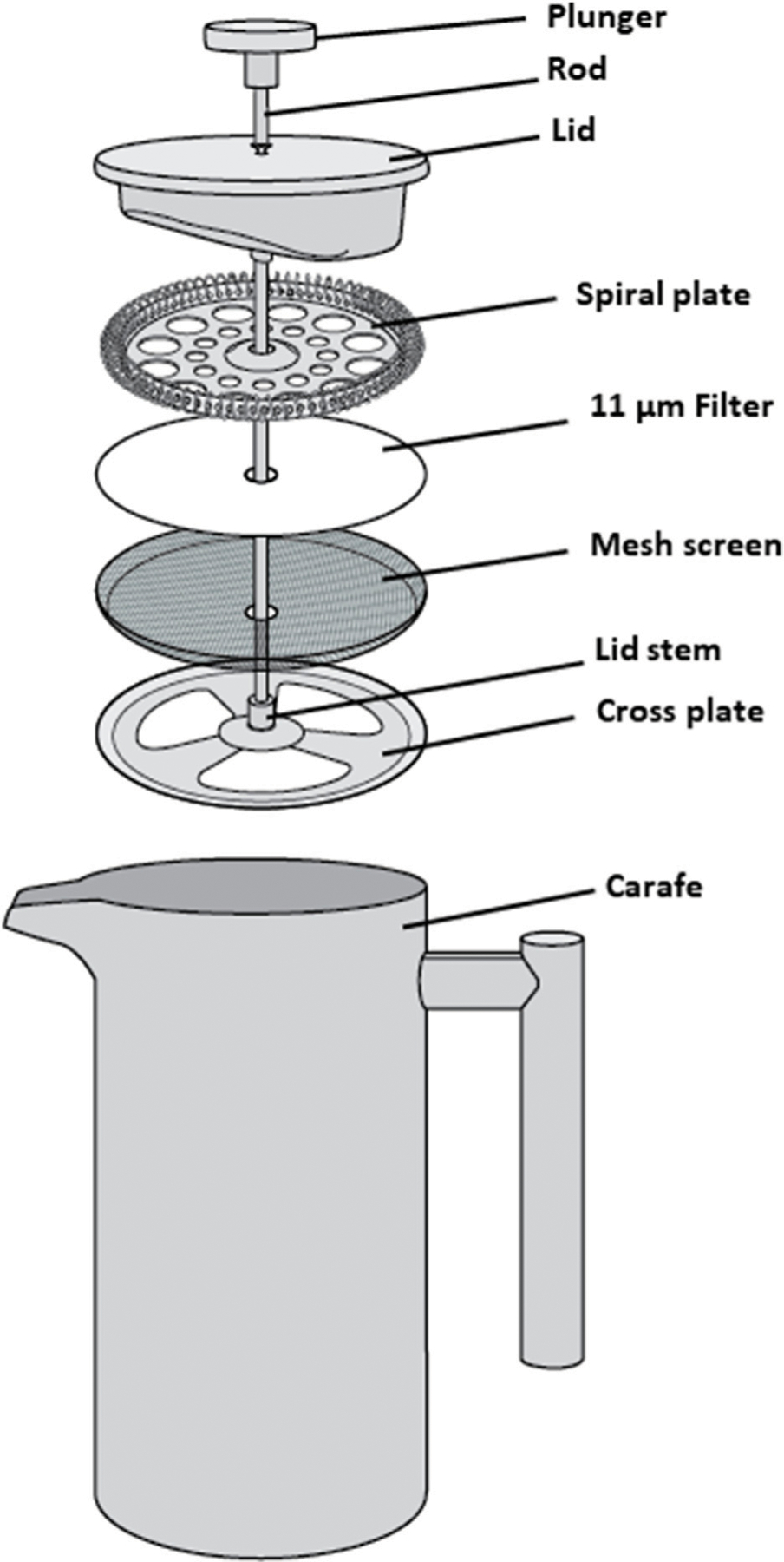
Schematic assembly of a 1 L stainless-steel coffee press, used to press wastewater samples.

**FIGURE 3 |:**
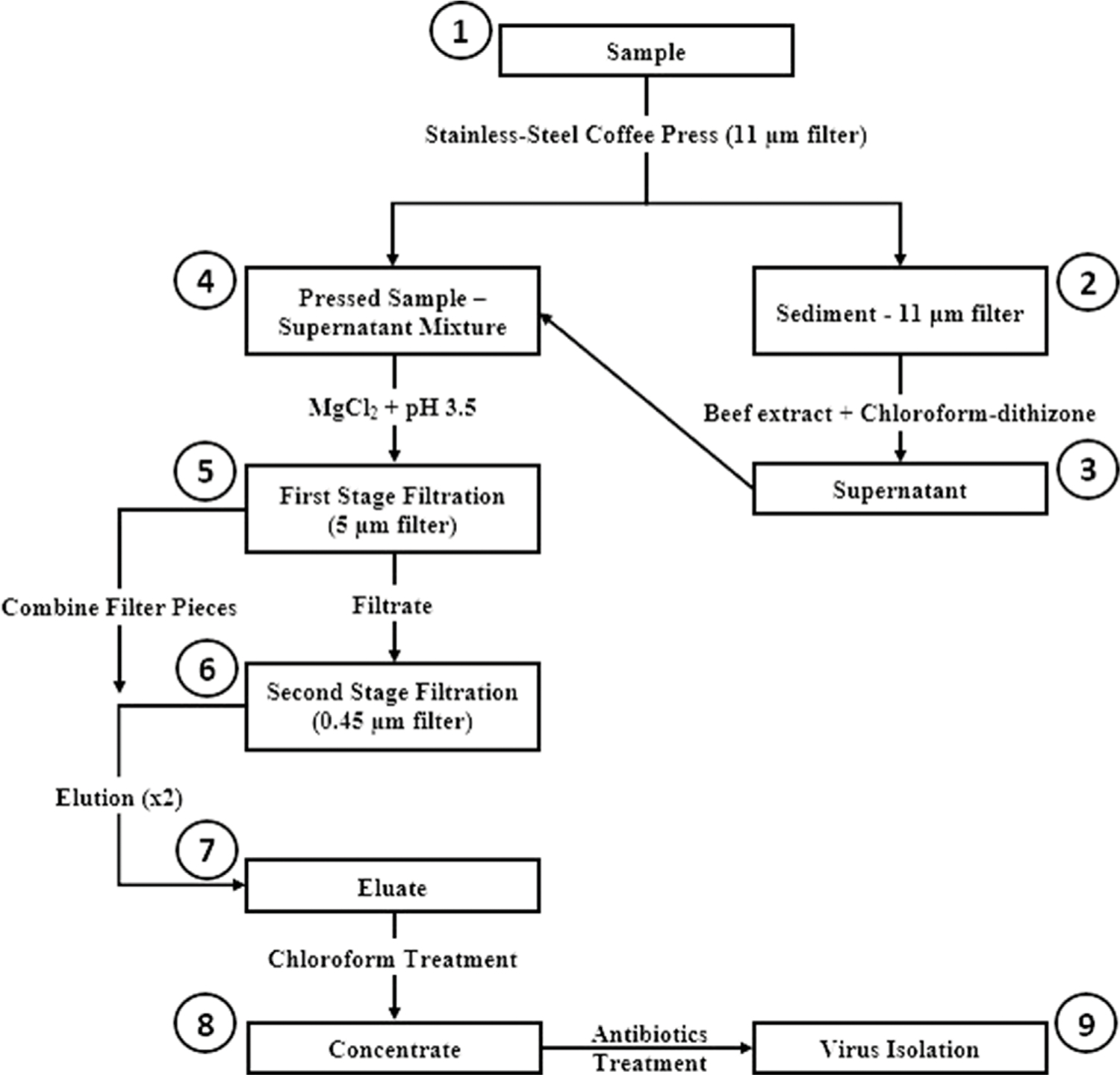
Concentration and Elution Filtration (CaFÉ) method workflow chart. 1) Sample is pressed using coffee press with 11 µm filter. 2) Viral particles are extracted from Sediment—11 µm filter with beef extract and chloroforrn-dithizone. 3) Supernatant is added to the main pressed sample. 4) MgCl_2_ is added to the pressed sample-supernatant mixture and pH is adjusted to 3.5. 5) Sample is filtered using 5 µm filter. 6) Filtrate is then, filtered again with 0.45 µm filter and filter pieces from the two filtration stages are combined and eluted with beef extract. 7) The resulted eluate is chloroform treated. 8) Concentrate is inoculated into RD and L20B cell lines after antibiotics treatment. 9) Inoculated samples are observed for 5 days post-inoculation, following WHO ENVS Virus Isolation Algorithm.

The CaFÉ method is based on the very known principle of charge-based methods, where electrostatic forces are instrumental in virus-filter interactions. A volume of 500 ml was processed using the CAFÉ procedure, which utilizes a 1-L, stainless-steel coffee press (Vonshef, London, United Kingdom) using 11 µm cellulose filter papers (85 mm, grade 1, Cytiva Life Sciences, Hillerød, Iceland) that is placed in between the spiral plate and the mesh screen (Figure 2). Wastewater was added to the carafe and then pressed to separate the heavy sediment from the liquid. As shown in Figure 3, viral particles were extracted with beef extract (3% w/v, pH 7.2 ± 0.2; Criterion, Hardy Diagnostics, Santa Maria, CA, United States) and chloroform-dithizone (0.001% w/v; Sigma-Aldrich) from the sediment and the 11 µm filter that were placed in a 50 ml conical tube, then agitated at 10,000 rpm for 10 min at room temperature (25°C) using Heidolph shaker (Schwabach, Germany) and centrifuged for 10 min at 1,500 x *g* at 4°C. The resulting supernatant was added to the main pressed sample. Magnesium chloride hexahydrate (2.5% w/v; EMD Millipore Corp, Burlington, MA, United States) was added to a final concentration of 0.05 M, and the pH was adjusted to 3.5 to facilitate and optimize virus adsorption to filter surfaces. The pressed sample-supernatant mixture was then passed through a series of two additional negatively charged mixed cellulose filters with a diameter of 47 mm (Advantec, Toyo Roshi Kaisha, Ltd., Uchisaiwaicho, Chiyoda City, Japan), using a vacuum pump (Cole-Parmer, IL, United States). If the sample is turbid with heavy sediment, 25 ml of the sample liquid is added at a time to the filter funnel. The first stage filtration uses a 5 µm filter, which captures virus particles aggregating to large clumps of sediment. During the second stage filtration, filtrate is then passed through a 0.45 µm filter, which captures virus particles aggregating to finer sediment. Both filters were subsequently cut into four pieces and placed in a 50 ml conical tube containing beef extract (3% w/v, pH 7.2 ± 0.2), which will slightly change the virus`s natural charge (or that of the filter) to facilitate virus elution from the filter, and agitated with heidolph shaker for 20 min, at 10,000 rpm at room temperature (25°C). The resulting eluate (15–18 ml) was treated with chloroform (20% v/v), agitated for 20 min at 10,000 rpm at room temperature (25°C), and then centrifuged at 1,500 x *g* for 20 min at 4°C. Antibiotics (100 IU/ml penicillin, 100 µg/ml streptomycin, and 50 µg/ml gentamycin) were added to the concentrate, then inoculated into cells for poliovirus isolation the same day.

### 2.3 Virus Isolation and Molecular Characterisation

Polio and other enteroviruses were isolated using the recommended WHO PV isolation protocol as described previously (Organization, 2009). Briefly 500 µl of the concentrate is added to 5 T25 cm^2^ flasks seeded with confluent monolayer of L20B cell line (recombinant murine cells that express human PV receptor) and one flask seeded with RD cell line (cells derived from human rhabdomyosarcoma), then incubated at 36°C for 5 days post inoculation. Flasks were observed daily and if characteristic enterovirus cytopathic effect (CPE) appears at any stage after inoculation (i.e., rounded, refractile cells detaching from the surface of the vessel), observation is recorded, and CPE is allowed to develop until at least 75% of the cells are affected (≥3 + CPE). At this stage, a second passage is performed by inoculating 500 µl of the supernatant CPE-positive cultures from the first passage in the opposite cell line (CPE positive L20B cultures were passaged into RD cell line and RD positive cultures were passaged into L20B cell line), This passage is aimed at separating polioviruses that may be present in mixtures with other enteroviruses and amplifying the titer of any polioviruses that may be present. CPE-positive cultures from the second passage of RD-L20B culture is passaged into a third passage into RD cell line and this passage is aimed at amplifying virus titer. To reduce bacterial contamination during virus isolation stage, antibiotics (200 IU/ml penicillin, 200 µg/ml streptomycin, and 50 µg/ml gentamycin) were added to the maintenance medium for every virus isolation passage.

Virus isolation is followed by the detection and differentiation of PV serotypes and genotypes with the GPLN intratypic differentiation (ITD) kit 5.0 (Organization, 2015a; Gerloff et al., 2018). Each ITD PCR mixture consists of 10 µl of qScript XLT one-step RT-quantitative PCR (qPCR) ToughMix (Quanta Biosciences, Beverly, MA), 1 µl of primer-probe mixture (contained in the ITD 5.0 kit; CDC, Atlanta, GA), 8 µl RNase-free water, and 1 µl of template (cell culture supernatant or extracted viral RNA). The thermocycling conditions are 30 min at 50°C for the RT step and 1 min of incubation at 95°C, followed by 40 cycles of 95°C for 15 s, 50°C for 1 min, and 72°C for 5 s (A reduced ramp rate of 25% between annealing and elongation was applied on the Applied Biosystems 7,500 real-time PCR system (Thermo Fisher Scientific).)

Viral protein 1 (VP1) sequencing was performed according to the WHO poliovirus testing algorithm. Sanger dideoxy-sequencing was done following previously described procedures (Kilpatrick et al., 2011; Burns et al., 2016). Briefly, RNA was extracted and reverse-transcribed before DNA amplification of the VP1 region of the capsid gene. The VP1 sequences were compared to Sabin reference strain sequences and nucleotide changes were analyzed using sequencher (5.4.6).

### 2.4 Statistical Analyses

Descriptive statistics were calculated using R software (R Core Team, 2016). The exact McNemar Chi square test with continuity correction was used to compare the results after isolation of enteroviruses and Sabin-like polioviruses from the two-phase separation and CaFÉ methods (McNemar, 1947; Fagerland et al., 2013) using the gmodels package (Trajman and Luiz, 2008). Data visualizations were made using ggplot2 package in R (Wickham, 2009).

## 3 RESULTS

### 3.1 Parallel and Pilot Testing

**FIGURE 4 |:**
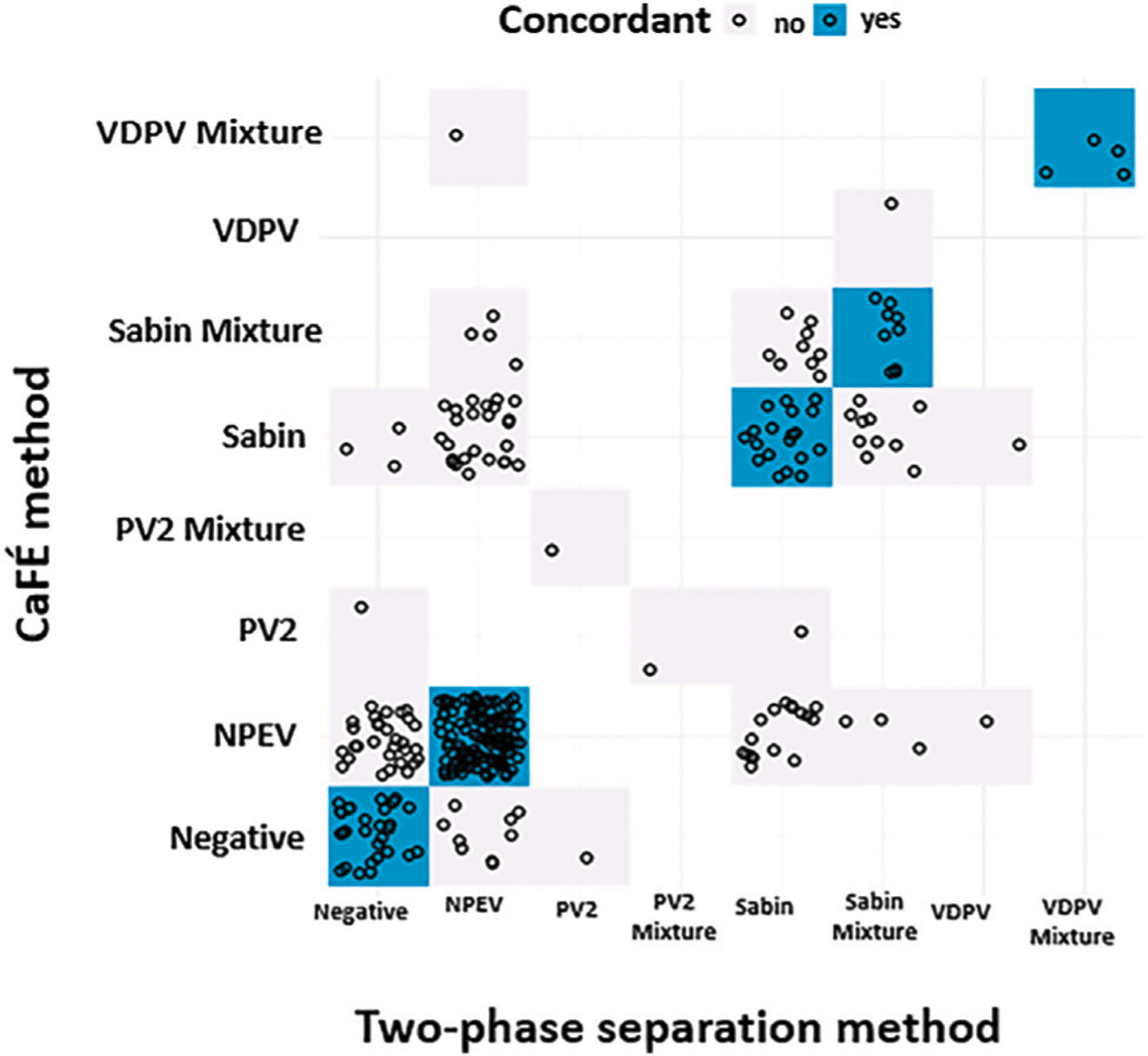
Poliovirus screening results forthe parallel and pilot studies between two-phase separation method and CaFÉ method by poliovirus genotype (n = 345), negative for poliovirus or mixture of multiple polioviruses. NPEV = non-poliovirus enterovirus, Sabin = Sabin-like type 1 or 3, PV2 = Poliovirus type 2, VDPV = Vaccine Derived poliovirustype 1, 2 or 3.

Concordance between the two methods for detecting any enterovirus occurred in 133 of 225 (59.1%) tested samples (Table 3 and Figure 4). There was no statistical difference between the two methods for the detection of Sabin-Like Polioviruses (SL PVs) (*p-value* > 0.05, McNemar) that were isolated from samples collected from Haiti, Guatemala, Thailand, Philippines, and Papua New Guinea in different months (Table 2). Overall, the two-phase separation method detected 21 poliovirus positive specimens that were not detected by the CaFÉ method, and the CaFÉ method, detected 36 more poliovirus positive samples than the two-phase separation method (Table 2; Figure 4). The sensitivity and the specificity of CaFÉ for the detection of poliovirus compared with the “gold standard” method (e.g., two-phase separation method) were 72.7 and 86.2%, respectively (Table 2). When comparing the detection of non-polio enteroviruses (NPEV), the two methods were significantly different (*p* > 0.0001, McNemar). The CaFÉ method detected NPEV in 46 samples that were reported as NPEV but were not detected by the two-phase method (Table 3; Figure 4). The sensitivity and the specificity of CaFÉ for the detection of NPEV compared with the two-phase separation method was 93 and 44% respectively (Table 3).

#### 3.1.1 Haïti

From April 2018 through November 2019, eight Sabin-Like type 3 polioviruses (SL 3 PVs) were isolated from samples collected from seven sites (Bois de chêne (BDC), Bois de Neuf (BNF), Boulevard de l`venir (BRA), Ruelle Caporis (CRC), Rue Pétion (PET), Route Rails Diquini (RRD) and Avenue Maurepas (AMA)). Sabin-Like type 1 polioviruses (SL 1 PVs) were only isolated from RRD on April 2018 and from BNF in December 2019, using the CaFÉ method. The same samples were NPEV or not detected with the two-phase separation method. The two-phase separation method detected four SL 3 PVs from four sites (BDC, RRD, AMA and PET), that were not detected using the CaFÉ method (Alleman et al., 2021a) (Supplementary Figure S1).

#### 3.1.2 Guatemala

In January 2019, VDPV1(Accession (ACC) no. MZ313559) was isolated from a sample collected from Aldea Cruz Blanca site (ACB) using the CaFÉ method, and VDPV3 (ACC.no. MZ313558) from a sample collected from Rio Platanitos site (PLA) in March 2019 using the two-phase separation method. Sequencing of the viral protein 1 (VP1) regions of the capsid protein showed 11 nucleotide differences compared to their respective Sabin reference for each one; therefore, both viruses met the definition of VDPV (Burns et al., 2014) (Supplementary Figure S2).

Two-phase separation method detected seven samples with a mixture of Sabin-Like type1 polioviruses (SL1 PVs) and SL3 PVs from ACB, Bodega Municipal (CMB) and Cuidad Quetzal (CQU) sites between November 2018—April 2019. Six of these parallel tested samples from the same collection sites were SL3 PVs with the CaFÉ method and one was NPEV (ACB site). Three samples with a mixture of SL1 PVs and SL3 PVs were isolated with the CaFÉ method from CMB and CQU sites. Two-phase separation method detected four SL3 PVs (CBM, CQU and PLA), that were NPEV with the CaFÉ method.

From November 2018—August 2019, the CaFÉ method detected four SL3 PVs that were NPEV with the two-phase separation method (ACB, Colinas de Villa Nueva (CVP) and PLA) (Supplementary Figure S2).

#### 3.1.3 Thailand

For the six sites that were chosen by NIH laboratory as part of their in-country poliovirus environmental surveillance, the CaFÉ method detected more SL PVs and NPEVs (Table 2 and 3) than the two-phase separation method. SL 1 PV was detected in five samples using the CaFÉ method from four collection sites (Din Deang, Nong Kheam, Mae Sot Hospital and Huay Mak Kaeng), whereas the two-phase separation method identified either NPEV or not detected for those same samples.

SL 3 PVs isolated from Nong Kheam and Nongsim sites and the mixture of both SL 1 PVs and SL 3 PVs in Nong Kheam were detected by the CaFÉ method but not by two-phase separation method (Supplementary Figure S3).

#### 3.1.4 The Philippines and Papua New Guinea

SL 2 PVs were isolated from samples collected in April and May 2020 at the Manila site (Tondo Sewage Pumping Plant (TSP)) and at Quezon City sampling site (East Avenue Sewage Treatment Plant (ESP)), by both concentration methods. In Manila (TSP site), cVDPV1 were isolated from samples collected in July and September 2019 with both methods. A single cVDPV2 positive sample was detected from the same collection site with the CaFÉ method in January 2020. In Papua New Guinea, mixtures of cVDPV1, SL 1 PVs and SL 3 PVs were detected with both methods in all three collection sites in Port Moresby (Gerehu Sewage Lagoon (GSL), Waigani Sewage Lagoon (WSL), Joyce Bay Treatment Plant (JTP)) in October 2018. cVDPV1 was isolated from GSL site in November 2018 using the CaFÉ method, whereas the result for the same sample was NPEV with the two-phase separation method. Also, SL 1 PVs and SL 3 PVs were detected in all collection sites in the Philippines and Papua New Guinea with a similar rate between CaFÉ and the two-phase separation method (Supplementary Figure S4).

## 4 DISCUSSION

The CaFÉ method was developed and evaluated as part of an effort to increase the effectiveness and robustness of environmental surveillance for polioviruses, and it was tested in three different laboratories in the Philippines, Thailand, and United States. Based upon the results presented here, there was no significant difference in poliovirus detection based on the 338 sample pairs that were available for comparison by the two-phase separation and the CaFÉ methods during the parallel and pilot tests in global laboratories (Table 2). As the GPEI expands and environmental surveillance is established in new countries at risk for poliovirus circulation, there is an increasing need to establish an additional concentration method that will reduce costs as well as turnaround time. This study is not the first attempt to increase the sensitivity of enteroviruses detection in wastewater. VIRADEL, a method that was developed for water samples with low turbidity and involves the adsorption of viral particles to the filter membrane through ionic interaction followed by elution with pH adjustment. However, it is time consuming, labor intensive and needs large sample volume (1–400 L) and requires a secondary concentration procedure to reduce the volume of eluate to enhance the sensitivity of detection (Falman, et al., 2019). Because the GPLN includes laboratories in low-and middle-income countries, procedures must be robust, and any method use internationally accessible supplies and reagents. The CaFÉ method uses less expensive reagents, bringing the average cost per sample to $10 US. The two-phase separation method costs $50 US/sample, with 2 days to process samples (Table 4), whereas the BMFS costs $100/sample and 2 days processing time (Fagnant et al., 2014; Fagnant et al., 2018; Zhou et al., 2018; Coulliette-Salmond et al., 2019). Processing time for CaFÉ is only 1 day, and all reagents and equipment are commonly available from global suppliers. even in low and middle-income countries.

The CaFÉ method verified the trend observed during the period of surveillance in Haïti (Coulliette-Salmond et al., 2019; Alleman et al., 2021a), where poliovirus detection occurred mostly after vaccine campaigns. Poliovirus detection was low, possibly indicating a low rate of poliovirus vaccination in Haiti during the study period. Some collection sites that were negative for any enteroviruses for consecutive months with the two-phase separation method (RRD, AMA, PET), had NPEV results with the CaFÉ method and were considered sufficiently sensitive sites, since detection of NPEV, is used as a proxy for PVs and ES site sensitivity (Kroiss et al., 2018). For an ES site to be considered sufficiently sensitive, the NPEV lower positive rate limit should be ≥50% for over 6-month period (Coulliette-Salmond et al., 2019) (Supplementary Figure S1).

The sites selected for Guatemala were adequate since both methods detected mixtures of SL1 PVs and SL 3 PVs in all sites from December 2018—August 2019. Relative to routine immunization, polio vaccination campaigns target high numbers of children in a wide age range (0–59 months of age) in a very concentrated period (2–3 days). Increases of Sabin viruses in wastewater are typically observed and expected during and just after supplementary immunization activities (SIAs) (Kroiss et al., 2018); however, environmental surveillance can also detect Sabin viruses administered through routine immunization when vaccine coverage rates are high enough. VDPV1 and VDPV3 were also detected in Guatemala. These VDPV isolates were not classified as circulating VDPV because there were no genetically linked viruses (Burns et al., 2014). The results of the six sites selected in Thailand showed that the CaFÉ method detected more positive results of SL1 PVs, SL3 PVs and mixtures of both compared to the two-phase separation method.

In the Philippines, VDPV1 and VDPV2 outbreaks were declared in September 2019, by January 2020, cVDPV2 was detected in an environmental sample in Manila, using the CaFÉ method and was later confirmed with an AFP case (Maklin et al., 2019). Both methods detected cVDPV1 outbreak in October 2018 in Papua New Guinea.

GPLN ES Guidelines recommend the concentration of at least 500 ml of wastewater and fecal-impacted environmental water samples when using the two-phase separation method. As the GPEI expands and environmental surveillance is established in new countries at risk for poliovirus circulation, there is an increasing need to establish an additional concentration method that will reduce costs as well as turnaround time.

Limitations for this study are samples consisting of thick sediment and large particulates, which resulted in clogged filters during the two filtration stages for the CaFÉ procedure. This was resolved by allowing the sediment to settle, followed by transferring 25 ml of the sample liquid to the filter funnel multiple times, which accelerated the filtration process for turbid samples. In addition, another limitation was the inherent variability of environmental samples and virus isolation in cell culture. The low frequency of PV detection in Haïti samples was another limitation, which reduced the statistical power for PV detection for both methods.

The CaFÉ method performed as well as the two-phase separation method and was able to detect Sabin-Like PVs and NPEVs in Guatemala, Haïti and Thailand and emerging VDPVs in the Philippines and Papua New Guinea. CaFÉ is a sensitive, cost-effective, and simple method to meet the needs for expanded environmental surveillance during the final stages of poliovirus eradication.

Future work should focus on exploring new settings for the CaFÉ method use, such as integrative approaches of environmental surveillance for multiple pathogens, since various methods—the skimmed milk flocculation and polyethylene glycol precipitation (PEG) as well as filtration methods (BMFS and ultrafiltration via Millipore filtration concentration) were used for SARS-CoV-2 surveillance in wastewater (Philo et al., 2021). This could also be an important next step for the CaFÉ method use to strengthen polio transition planning and sustain detection capabilities for long-term.

## Supplementary Material

Supplemental materials

## Figures and Tables

**TABLE 1 |:** Poliovirus environmental surveillance sites used during the parallel and pilot testing.

Country	City	Site Code*	Geographic Coordinates	Estimated Watershed Population	Additional Site Details
**Haïti**	Port au prince	BNF	18.5815, −72.3291	347,237	Coulliette-Salmond et al. (2019); Alleman et al. (2021a)
		BDC	18.5383, −72.3539	339,624	Coulliette-Salmond et al. (2019)
		RRD	18.5345, −72.3842	67,320	Alleman et al. (2021a)
	Gonaïves	BRA	19.4383, −72.6895	20,241	Alleman et al. (2021a)
		KHF	19.4534, −72.6900	25,703	Coulliette-Salmond et al. (2019)
	Saint Marc	AMA	19.1059, −72.6983	50,744	Alleman et al. (2021a)
		PET	19.1101, −72.6983	49,372	Alleman et al. (2021a)
	Cap Haïtian	HUC	19.1224, −72.6978	5,655	Alleman et al. (2021a)
		GRC	19.7367, −72.2155	5,455	Alleman et al. (2021a)
		CRC	19.7335, −72.2178	113,023	Alleman et al. (2021a)
		RPA	19.7383, −72.1843	22,468	Alleman et al. (2021a)

**Guatemala**	San Juan	CQU	14.7166, −90.5626	2,643	Open canal
	Sacatepéquez	CBM	14.7236–90.6520	1,597	Open canal
		ACB	14.7283, −90.5829	8,726	Open canal
	Villa Nueva	PLA	14.5104, 14.5104	164,963	Open canal
		CVP	14.5733, −90.5893	81,264	Open canal
		PPT	14.5063, −90.5893	6,992	Wastewater treatment plant

**Thailand**	Bangkok	NKM	13.7207–100.3564	55,000	Water environment control plant (closed system)
		DIN	13.7721–100.5583	1,080,000	Water environment control plant (closed system)
	Udonthani	UD-A	17.4203–102.8026	800,000	Wastewater pumping station (opened system)
		UD-B	17.4365–102.8026	800,000	Wastewater pumping station (opened system)
	Tak	MS-A	16.7109–98.5722	101,000	Hospital wastewater treatment plant (closed system)
		MS-B	16.7144–98.5515	103,000	Wastewater treatment plant (opened system)

**Philippines**	Quezon City	ESP	14.6458–121.0449	74,000	Wastewater treatment plant. It treats 16 million liters/day of domestic wastewater
	Manila	TSP	14.6028–120.9668	631,363	Wastewater treatment plant. It has a capacity of 432,000 cubic meter/day, covering 70% of Manila
	Benguet	BAG	16.4296–120.5975	345,366	Wastewater treatment plant. It treats only sewage from the Balili catchment area

**Papua New Guinea**	Port Moresby	GSL	−9.3736–147.1641	391,000	Open sewage Lagoon, covering the catchment areas of Vanapa and Brown rivers
	—	WSL	−9.3897–147.1983	391,000	Pond, where Boroko creek drains into it
	—	JTP	−9.4991–147.1922	391,000	Wastewater treatment plant. Treats 18.4 million liters per day, comprises 26 km and 1.2 k ocean outfall

* Port au Prince: BNF, Bois de Neuf; BDC, Bois de Chêne; RRD, Route Rails Diquini. Gonaïves: BRA, Boulevard de l`venir, KHF, Key Soleil Health Facility. Saint Marc: AMA, Avenue Maurepas; PET, Rue Pétion; HUC, Impass Hucar. Cap-Haïtien: GRC, Grand rue Champin; CRC, Ruelle Caporis; RPA, Ruelle Patience. San Juan Sacatepéquez: CQU, Cuidad Quetzal; CBM, Bodega Municipal; ACB, Aldea Cruz Blanca. Villa Nueva: PLA, Rio Platanitos; CVP, Colinas de Villa Nueva; PPT, Peronia Planta de Tratamiento de Auga. Bangkok: NKM, Nongkheam; DIN, Dindeang. Udonthani: UD-A, Nongsim; UD-B, Huay Mak Khaeng. Tak: MS-A, Mea Sot Hospital; MS-B, Municipality Nakorn Mae Sot. Quezon City: ESP, East Avenue Sewage Treatment Plant. Manila: TSP, Tondo Sewage Pumping Plant. Benguet: BAG, Baguio Sewage Treatment Plant. Port Moresby: GSL, Gerehu Sewage Lagoon; WSL, Waigani Sewage Lagoon; JTP, Joyce Bay Treatment Plant.

**TABLE 2 |:** Comparison between the two-phase separation method and concentration and filtration elution (CaFÉ) method for the isolation of any Sabin-like poliovirus during the parallel and pilot tests: Haïti (December 2017—December 2019), Guatemala (November 2018—August 2019), Thailand (February 2019—July 2019), the Philippines (October 2018—December 2020) and Papua New Guinea (October 2018—December 2019).

	Positive Two-phase	Negative Two-phase	Total
	Separation	Separation	
Positive CaFÉ	56	36	92
Negative CaFÉ	21	225	246
Total	77	261	338

*CaFÉ, concentration and filtration elution*. p-*value* > *0.05. Sensitivity ((CI) = 72.7%) and specificity ((CI) = 86.2%), were not statistically significant as determined by the exact McNemar`s test (*p-*value* > *0.05).*

**TABLE 3 |:** Comparison between the two-phase separation method and concentration and filtration elution (CaFÉ) method, for the isolation of non-polio enterovirus during the parallel and pilot tests: Haïti (December 2017—December 2019), Guatemala (November 2018—August 2019), Thailand (February 2019—July 2019), the Philippines (October 2018—December 2020) and Papua New Guinea (October 2018—December 2019).

	Positive Two-phase	Negative Two-phase	Total
	Separation	Separation	
Positive CaFÉ	133	46	179
Negative CaFÉ	10	36	46
Total	143	82	225

*CaFÉ, concentration and filtration elution.* p-*value* < *0.0001. Sensitivity ((CI) = 93%) and specificity ((CI) = 44%), were statistically significant as determined by the exact McNemar`s test (*p-*value* < *0.0001).*

**TABLE 4 |:** Comparison of features between the two-phase separation method and concentration and filtration elution (CaFÉ) method.

	CaFÉ	Two-phase Separation
Processing time	1 day	2 days
Consumable cost	$10 US	$50 US
Sample volume	500 ml	500 ml
Reagents used and their shelf-life	Beef extract Solution: 3 months	Dextran T40: 2 weeks
	Magnesium chloride hexahydrate: N/A^a^	Polyethylene glycol 6,000: 2 weeks
	Chloroform-dithizone: 1 month	Sodium chloride: N/A
Centrifuge	Yes	Yes: with rotor capacity to hold 250 ml bottles

a Not applicable, these reagents have no shelf-life expiration date.

## Data Availability

The original contributions presented in the study are included in the article/Supplementary Material, further inquiries can be directed to the corresponding author.

## References

[R1] Alleman MM, Jorba J, Greene SA, Diop OM, Iber J, Tallis G, (2020). Update on Vaccine-Derived Poliovirus Outbreaks - Worldwide, July 2019- February 2020. MMWR Morb. Mortal. Wkly. Rep 69, 489–495. doi:10.15585/mmwr.mm6916a1 32324719 PMC7188410

[R2] Alleman MM, Coulliette-Salmond AD, Wilnique P, Belgasmi-Wright H, Sayyad L, Wong K, (2021a). Environmental Surveillance for Polioviruses in Haïti (2017–2019): The Dynamic Process for the Establishment and Monitoring of Sampling Sites. Viruses 13, 505. doi:10.3390/v13030505 33803868 PMC8003210

[R3] Alleman MM, Jorba J, Henderson E, Diop OM, Shaukat S, Traoré MA, (2021b). Update on Vaccine-Derived Poliovirus Outbreaks - Worldwide, January 2020-June 2021. MMWR Morb. Mortal. Wkly. Rep 70, 1691–1699. doi:10.15585/mmwr.mm7049a1 34882653 PMC8659190

[R4] Asghar H, Diop OM, Weldegebriel G, Malik F, Shetty S, El Bassioni L, (2014). Environmental Surveillance for Polioviruses in the Global Polio Eradication Initiative. J. Infect. Dis 210, S294–S303. doi:10.1093/infdis/jiu384 25316848 PMC10578309

[R5] Bigouette JP, Wilkinson AL, Tallis G, Burns CC, Wassilak SGF, and Vertefeuille JF (2021). Progress toward Polio Eradication - Worldwide, January 2019-June 2021. MMWR Morb. Mortal. Wkly. Rep 70, 1129–1135. doi:10.15585/mmwr.mm7034a1 34437527 PMC8389387

[R6] Blake IM, Pons-Salort M, Molodecky NA, Diop OM, Chenoweth P, Bandyopadhyay AS, (2018). Type 2 Poliovirus Detection after Global Withdrawal of Trivalent Oral Vaccine. N. Engl. J. Med 379, 834–845. doi:10.1056/NEJMoa1716677 30157398 PMC5985919

[R7] Burns CC, Diop OM, Sutter RW, and Kew OM (2014). Vaccine-derived Polioviruses. J. Infect. Dis 210, S283–S293. doi:10.1093/infdis/jiu295 25316847

[R8] Burns CC, Kilpatrick DR, Iber JC, Chen Q, and Kew OM (2016). “Molecular Properties of Poliovirus Isolates: Nucleotide Sequence Analysis, Typing by PCR and Real-Time RT-PCR,”. Poliovirus: Methods and Protocols, Methods in Molecular Biology Editor Martin J, 1387, 177–212. doi:10.1007/978-1-4939-3292-4_9 26983735

[R9] Coulliette-Salmond AD, Alleman MM, Wilnique P, Rey-Benito G, Wright HB, Hecker JW, (2019). Haiti Poliovirus Environmental Surveillance. Am. J. Trop. Med. Hyg 101, 1240–1248. doi:10.4269/ajtmh.19-0469 31701857 PMC6896891

[R10] El Bassioni L, Barakat I, Nasr E, De Gourville EM, Hovi T, Blomqvist S, (2003). Prolonged Detection of Indigenous Wild Polioviruses in Sewage from Communities in Egypt. Am. J. Epidemiol 158 (8), 807–815. doi:10.1093/aje/kwg202 14561671

[R11] Esteves-Jaramillo A, Estivariz CF, Penaranda S, Richardson VL, Reyna J, Coronel DL, (2014). Detection of Vaccine-Derived Polioviruses in Mexico Using Environmental Surveillance. J. Infect. Dis 210, S315–S323. doi:10.1093/infdis/jiu183 25316850 PMC10389690

[R12] Estívariz CF, Pérez-Sánchez EE, Bahena A, Burns CC, Gary HE Jr, García-Lozano H, (2019). Field Performance of Two Methods for Detection of Poliovirus in Wastewater Samples, Mexico 2016–2017. Food Environ. Virol 11, 364–373. doi:10.1007/s12560-019-09399-9 31571037 PMC10389298

[R13] Fagerland MW, Lydersen S, and Laake P (2013). The McNemar Test for Binary Matched-Pairs Data: Mid-p and Asymptotic Are Better Than Exact Conditional. BMC Med. Res. Methodol 13, 13–91. doi:10.1186/1471-2288-13-91 23848987 PMC3716987

[R14] Fagnant CS, Beck NK, Yang M-F, Barnes KS, Boyle DS, and Meschke JS (2014). Development of a Novel Bag-Mediated Filtration System for Environmental Recovery of Poliovirus. J. Water Health 12, 747–754. doi:10.2166/wh.2014.032 25473984

[R15] Fagnant CS, Sánchez-Gonzalez LM, Zhou NA, Falman JC, Eisenstein M, Guelig D, (2018). Improvement of the Bag-Mediated Filtration System for Sampling Wastewater and Wastewater-Impacted Waters. Food Environ. Virol 10, 72–82. doi:10.1007/s12560-017-9311-7 28674934 PMC5823955

[R16] Fagnant-Sperati CS, Ren Y, Zhou NA, Komen E, Mwangi B, and Hassa J (2020). Validation of the Bag-Mediated Filtration System for Environemental Surveillance of Poliovirus in Nairobi, Kenya. J App Microb 130, 971–981. doi:10.1111/jam.14807 PMC7854911 32743931

[R17] Falman JC, Fagnant-Sperati CS, Kossik AL, Boyle DS, and Meschke JS (2019). Evaluation of Secondary Concentration Methods for Poliovirus Detection in Wastewater. Food Environ. Virol 11, 20–31. doi:10.1007/s12560-018-09364-y 30612304 PMC6394643

[R18] Fomban leke RG, King A, Pallansch MA, Tangermann RH, Mkanda P, Chunsuttiwat S, (2020). Certifying the Interruption of Wild Poliovirus Transmission in the WHO African Region on the Turbulent Journey to a Polio-free World. Lancet Glob. Health 8, e1345–e1351. doi:10.1016/s2214-109x(20)30382-x 32916086 PMC7525084

[R19] Gerloff N, Sun H, Mandelbaum M, Maher C, Nix WA, Zaidi S, (2018). Diagnostic Assay Development for Poliovirus Eradication. J. Clin. Microbiol 56, e01624–17. doi:10.1128/JCM.01624-17 29212703 PMC5786708

[R20] Greene SA, Ahmed J, Datta SD, Burns CC, Quddus A, Vertefeuille JF, (2019). Progress toward Polio Eradication - Worldwide, January 2017- March 2019. MMWR Morb. Mortal. Wkly. Rep 68, 458–462. doi:10.15585/mmwr.mm6820a3 31120868 PMC6532951

[R21] Hovi T, Stenvik M, Partanen H, and Kangas A (2001). Poliovirus Surveillance by Examining Sewage Specimens. Quantitative Recovery of Virus after Introduction into Sewerage at Remote Upstream Location. Epidemiol. Infect 127, 101–106. doi:10.1017/s0950268801005787 11561962 PMC2869716

[R22] Hovi T, Blomqvist S, Nasr E, Burns CC, Sarjakoski T, Ahmed N, (2005). Environmental Surveillance of Wild Poliovirus Circulation in Egypt-balancing between Detection Sensitivity and Workload. J. Virological Methods 126, 127–134. doi:10.1016/j.jviromet.2005.02.002 15847928

[R23] Iwai M, Yoshida H, Matsuura K, Fujimoto T, Shimizu H, Takizawa T, (2006). Molecular Epidemiology of Echoviruses 11 and 13, Based on an Environmental Surveillance Conducted in Toyama Prefecture, 2002–2003. Appl. Environ. Microbiol 72, 6381–6387. doi:10.1128/AEM.02621-05 16957267 PMC1563678

[R24] Hovi T, Shulman LM, Van Der Avoort H, Deshpande J, Roivainen M, and De Gourville EM (2012). Role of Environmental Poliovirus Surveillance in Global Polio Eradication and beyond. Epidemiol. Infect 140, 1–13. doi:10.1017/s095026881000316x 21849095

[R25] Kalkowska DA, Franka R, Higgins J, Kovacs SD, Forbi JC, Wassilak SGF, (2020). Modeling Poliovirus Transmission in Borno and Yobe, Northeast Nigeria. Risk Anal 41, 289–302. doi:10.1111/risa.13485 32348621 PMC7814397

[R26] Kilpatrick DR, Iber JC, Chen Q, Ching K, Yang S-J, De L, (2011). Poliovirus Serotype-specific VP1 Sequencing Primers. J. Virological Methods 174, 128–130. doi:10.1016/j.jviromet.2011.03.020 21440569

[R27] Kroiss SJ, Ahmadzai M, Ahmed J, Alam MM, Chabot-Couture G, Famulare M, (2018). Assessing the Sensitivity of the Polio Environmental Surveillance System. PLoS ONE 13, e0208336. doi:10.1371/journal.pone.0208336 30592720 PMC6310268

[R28] Macklin G, Diop OM, Humayun A, Shahmahmoodi S, El-Sayed ZA, Triki H, (2019). Update on Immunodeficiency-Associated Vaccine-Derived Polioviruses - Worldwide, July 2018-December 2019. MMWR Morb. Mortal. Wkly. Rep 69, 913–917. doi:10.15585/mmwr.mm6928a4 PMC7366852 32673297

[R29] McNemar Q (1947). Note on the Sampling Error of the Difference between Correlated Proportions or Percentages. Psychometrika 12, 153–157. doi:10.1007/bf02295996 20254758

[R30] Nakamura T, Hamasaki M, Yoshitomi H, Ishibashi T, Yoshiyama C, Maeda E, (2015). Environmental Surveillance of Poliovirus in Sewage Water Around the Introduction Period for Inactivated Polio Vaccine in Japan. Appl. Environ. Microbiol 81, 1859–1864. doi:10.1128/aem.03575-14 25556189 PMC4325164

[R31] Ndiaye AK, Diop PA, and Diop OM (2014). Environmental Surveillance of Poliovirus and Non-polio Enterovirus in Urban Sewage in Dakar, Senegal (2007–2013). Pan Afr. Med. J 19, 243. doi:10.11604/pamj.2014.19.243.3538 25848458 PMC4377292

[R32] Novel-t, P. A. T. H. (2021). Environmental Surveillance, Supporting Polio Eradication Available online: https://es.world/.

[R33] Organization, W. H. (1988). Global Eradication of Poliomyelitis by the Year 2000 Geneva: World Health Organization. Available at: https://apps.who.int/iris/bitstream/handle/10665/66495/WHO_POLIO_00.05.pdf.

[R34] Organization, W. H. (2003). Guidelines for Environmental Surveillance of Poliovirus Circulation Geneva: World Health Organization. biologicals Available at: https://pesquisa.bvsalud.org/portal/resource/pt/who-67854.

[R35] Organization, W. H. (2009). Supplemental to the WHO Polio Laboratory Manual: An Alternative Test Algorithm for Poliovirus Isolation and Characterization Geneva: World Health Organization. Available at: https://polioeradication.org/wpcontent/uploads/2017/05/NewAlgorithmForPoliovirusIsolationSu-pplement1.pdf.

[R36] Organization, W. H. (2015a). Guidelines on Environmental Surveillance for Detection of Polioviruses Geneva: World Health Organization. Available at: http://polioeradication.org/wp-content/uploads/2016/07/GPLN_GuidelinesES_April2015.pdf.

[R37] Organization, W. H. (2015b). Polio Environmental Surveillance Expansion Plan-Global Expansion Plan under the Endgame Strategy 2013–2018 Geneva: World Health Organization.

[R38] Organization, W. H. (2015c). Global Polio Eradication Initiative. Data and Monitoring Geneva: World Health Organization. Available at: http://www.polioeradication.org/dataandmonitoring.aspx.

[R39] Organization, W. H. (2019). The Polio Endgame Strategy 2019–2023: Eradication, Integration, Containment, and Certification Geneva: World Health Organization. Available at: http://polioeradication.org/wp-content/uploads/2019/06/english-polio-endgame-strategy.pdf.

[R40] Organization, W. H. (2021). Polio Eradication Strategy 2022–2026: Delivering on a Promise Geneva: World Health Organization. Available at: https://polioeradication.org/wp-content/uploads/2021/06/polio-eradication-new-Strategy-2022-26-Executive-Summary.pdf.

[R41] Organization, W. H. (2022). Disease Outbreak News; Wild Poliovirus Type 1 (WPV1) – Malawi Geneva: World Health Organization. Available at: https://www.who.int/emergencies/disease-outbreak-news/item/wild-poliovirus-type-1-(WPV1)-malawi.

[R42] Philo SE, Keim EK, Swanstrom R, Ong AQW, Burnor EA, Kossik AL, (2021). A Comparison of SARS-CoV-2 Wastewater Concentration Methods for Environmental Surveillance. Sci. Total Environ 760, 144215. doi:10.1016/j.scitotenv.2020.144215 33340739 PMC7832770

[R43] Pöyry T, Stenvik M, and Hovi T (1988). Viruses in Sewage Waters during and after a Poliomyelitis Outbreak and Subsequent Nationwide Oral Poliovirus Vaccination Campaign in Finland. Appl. Environ. Microbiol 54, 371–374. doi:10.1128/aem.54.2.371-374.1988 2833160 PMC202459

[R44] R Core Team (2016). R: A Language and Environment for Statistical Computing, v3.3.1 Vienna, Austria: R Foundation for Statistical Computing.

[R45] Trajman A, and Luiz RR (2008). McNemar χ2test Revisited: Comparing Sensitivity and Specificity of Diagnostic Examinations. Scand. J. Clin. Laboratory Investigation 68, 77–80. doi:10.1080/00365510701666031 18224558

[R46] Wickham H (2009). ggplot2: Elegant Graphics for Data Analysis New York, NY: Springer Science+Business Media.

[R47] Zhou NA, Fagnant-Sperati CS, Shirai JH, Sharif S, Zaidi SZ, Rehman L, (2018). Evaluation of the Bag-Mediated Filtration System as a Novel Tool for Poliovirus Environmental Surveillance: Results from a Comparative Field Study in Pakistan. PLoS One 13, e0200551–S97. doi:10.1371/journal.pone.0200551 30011304 PMC6047795

