# Supplementary material for: CaFÉ: A Sensitive, Low-Cost Filtration Method for Detecting Polioviruses and Other Enteroviruses in Residual Waters
Source: Front Environ Sci. Author manuscript; Available in PMC 2023 Jul 4. (PMC9344547; doi:10.3389/fenvs.2022.914387)
Supplement: Supplemental materials [file NIHMS1821388-supplement-Supplemental_materials.pdf]

frontiers

***Supplementary Material***

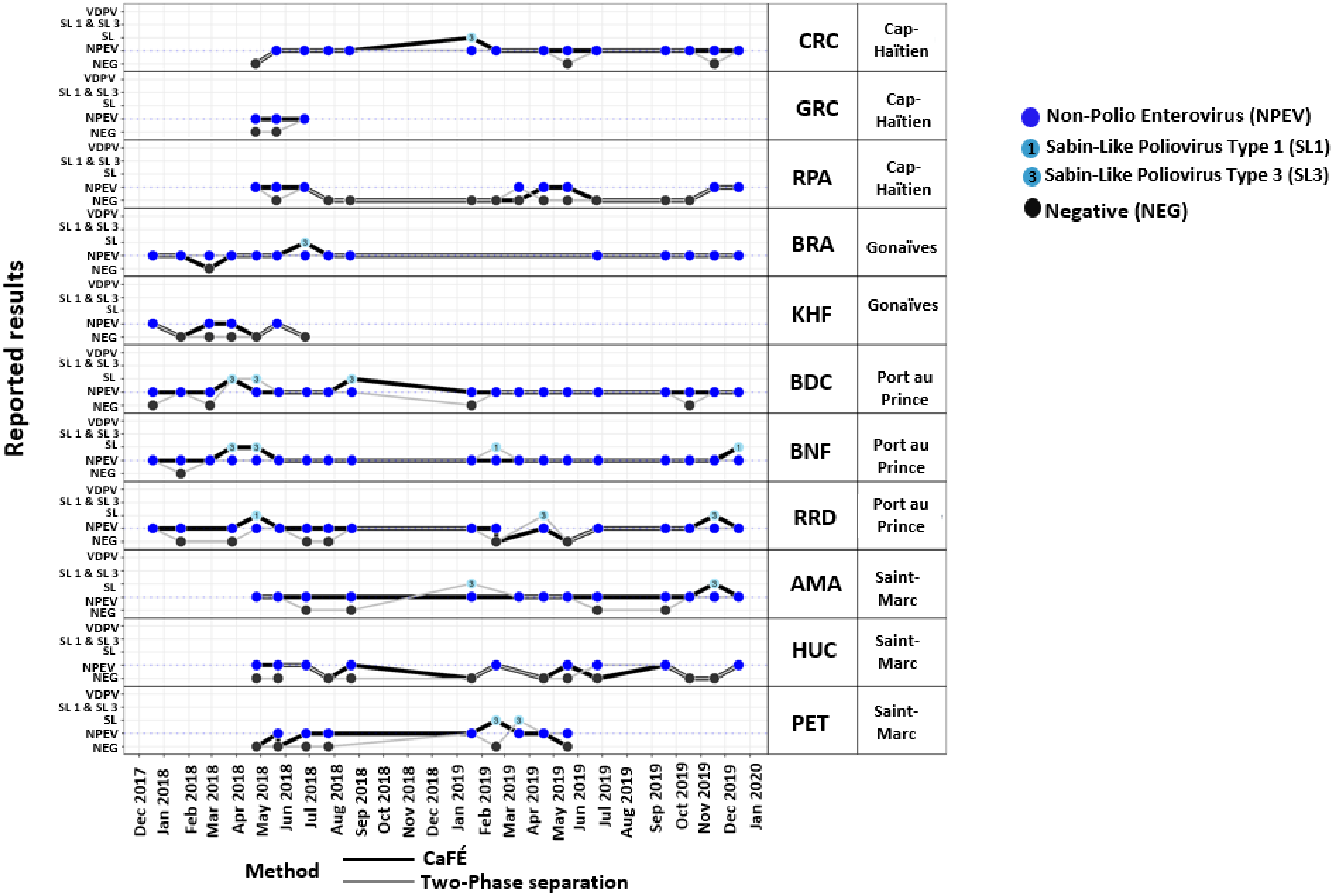


**Supplementary Figure 1.** Enteroviruses isolated through Haiti parallel testing for the CaFÉ (Concentration and Elution Filtration) method and the two-phase separation method, during December 2017 - December 2019, by month, year, sampling sites (Cap Haïtien: CRC = Ruelle

Caporis, GRC = Grand Rue, RPA = Ruelle patience. Gonaïves : BRA = Boulevard de l`Avenir, KHF = Key Soleil Health Facility. Port au Prince : BDC = Bois de chêne, BNF = Bois de Neuf, RRD = Route Rails Diquini. Saint Marc: AMA = Avenue Maurepas, HUC = Impass Hucar, PET = Rue Pétion), reported results, and the sample concentration method (two-phase separation and CaFÉ).● NPEV = Non-Polio Enterovirus. ① SL1 = Sabin-Like Poliovirus Type 1. ③ SL3 = Sabin-Like Poliovirus Type 3. ● NEG = Negative. (Alleman et al., 2021a)

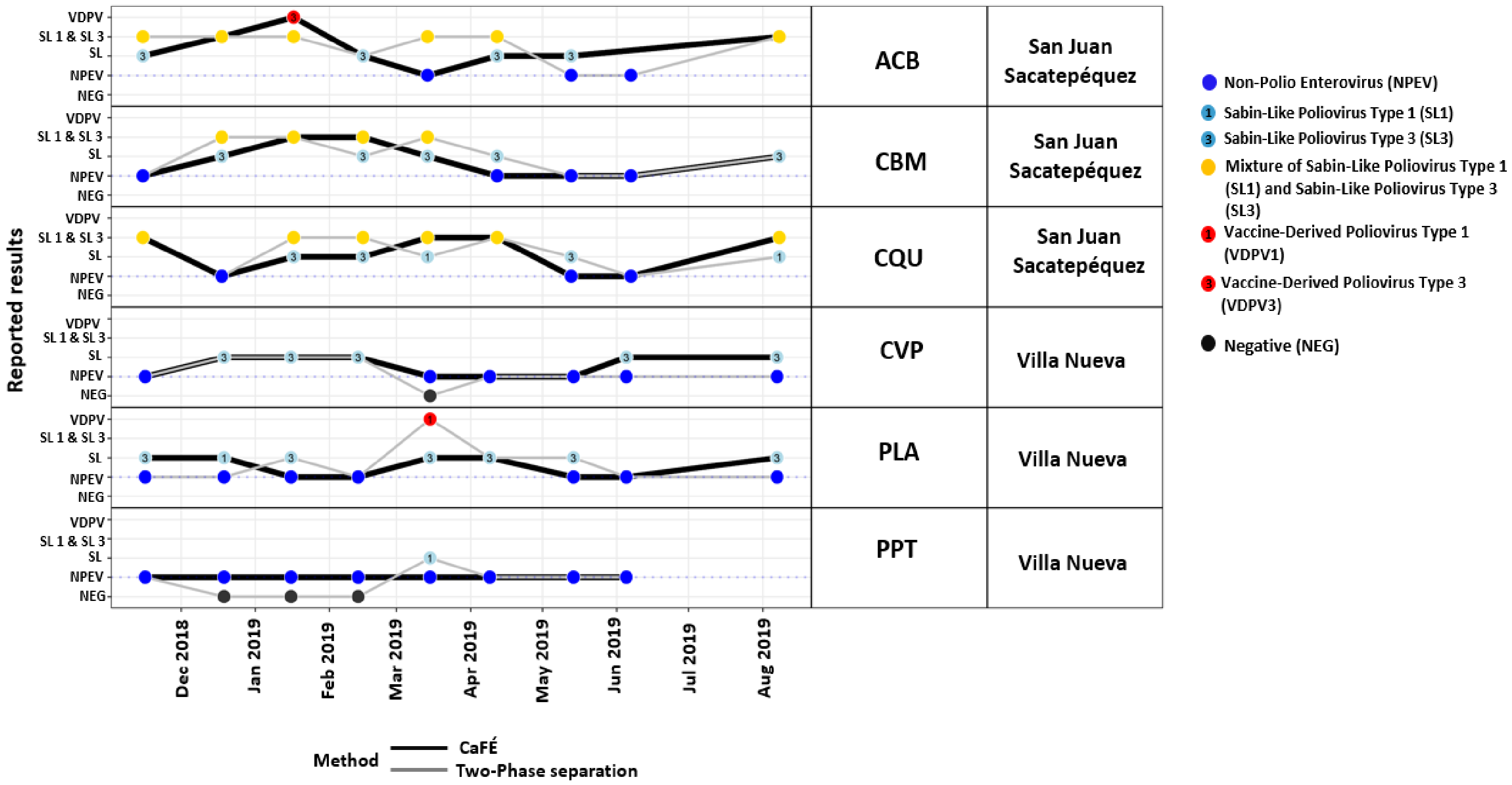


**Supplementary Figure 2.** Enteroviruses isolated through Guatemala parallel testing for the CaFÉ method**,** during November 2018 - August 2019 by month, year, sampling sites (San Juan Sacatepéquez: ACB = Aldea Cruz Blanca, CBM= Bodega Municipal, CQU = Cuidad Quetzal. Villa Nueva: CVP = Colinas de Villa Nueva, PLA = Rio Platanitos, PPT = Peronia Planta de Tratamiento de Auga), reported results, and the sample concentration method (two-phase separation and CaFÉ). ● NPEV = Non-Polio Enterovirus. ① SL1 = Sabin-Like Poliovirus Type 1. ③ SL3 = Sabin-Like Poliovirus Type 3. ● SL1 & SL3 = Mixture of Sabin-Like Poliovirus Type 1 and Sabin-Like Poliovirus Type 3. ① VDPV1 = Vaccine-Derived Poliovirus Type 1. ③ VDPV3 = Vaccine-Derived Poliovirus Type 3. ● NEG = Negative.

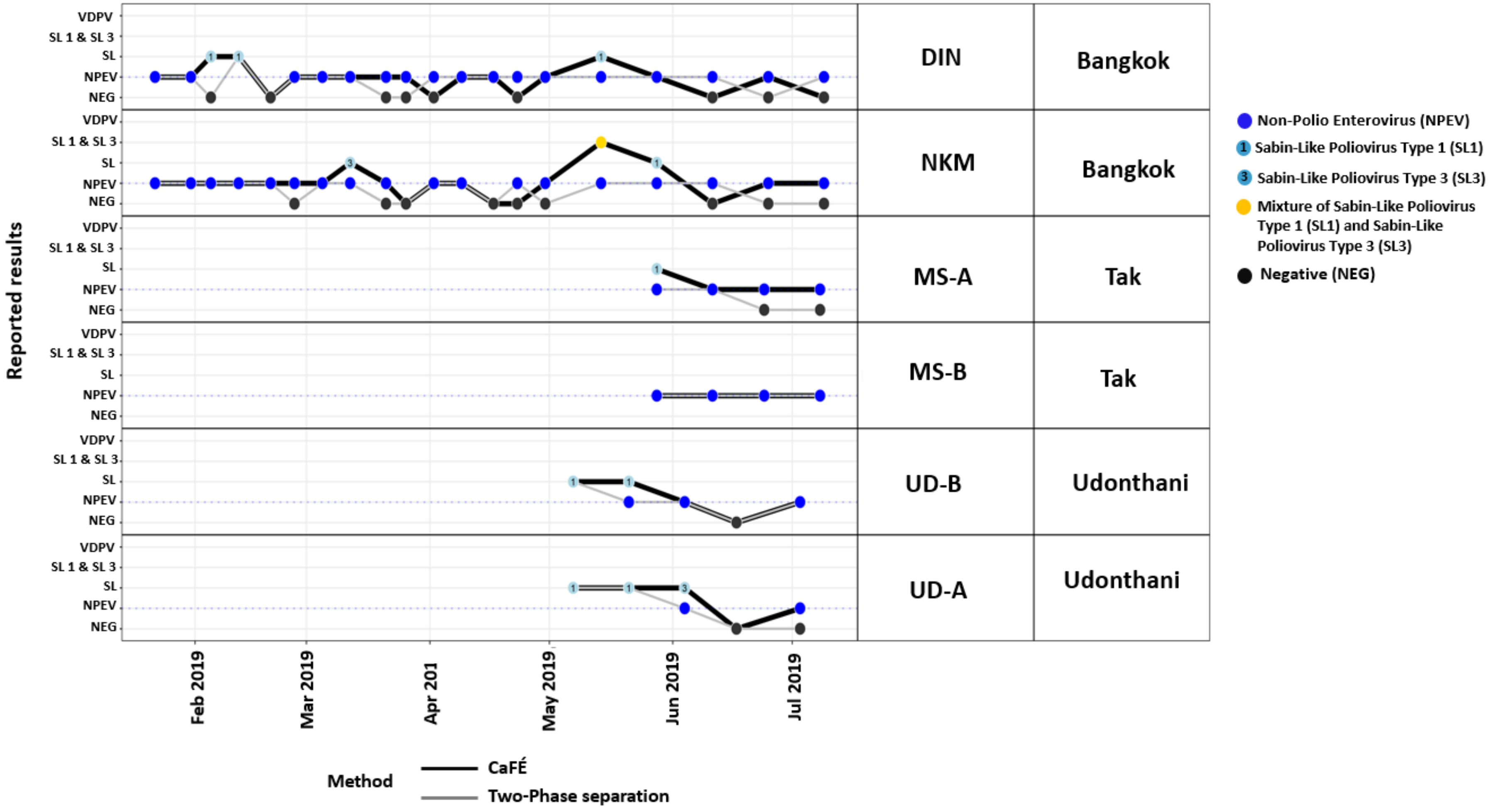


**Supplementary Figure 3.** Enteroviruses isolated through Thailand pilot testing for the CaFÉ method during February 2019 - July 2019 by month, year, sampling sites (Bangkok: DIN = Din Daeng, NKM = Nong Kheam. Tak: MS-A = Mea Sot hospital, MS-B = Municipality Nakorn Mae Sot. Udonthani: UD-B = Huay Mak Khaeng, UD-A= Nongsim), reported results, and the sample concentration method (two-phase separation and CaFÉ). ● NPEV = Non-Polio Enterovirus. ① SL1 = Sabin-Like Poliovirus Type 1. ③ SL3 = Sabin-Like Poliovirus Type 3. ● SL1 & SL3 = Mixture of Sabin-Like Poliovirus Type 1 and Sabin-Like Poliovirus Type 3. ● NEG = Negative.

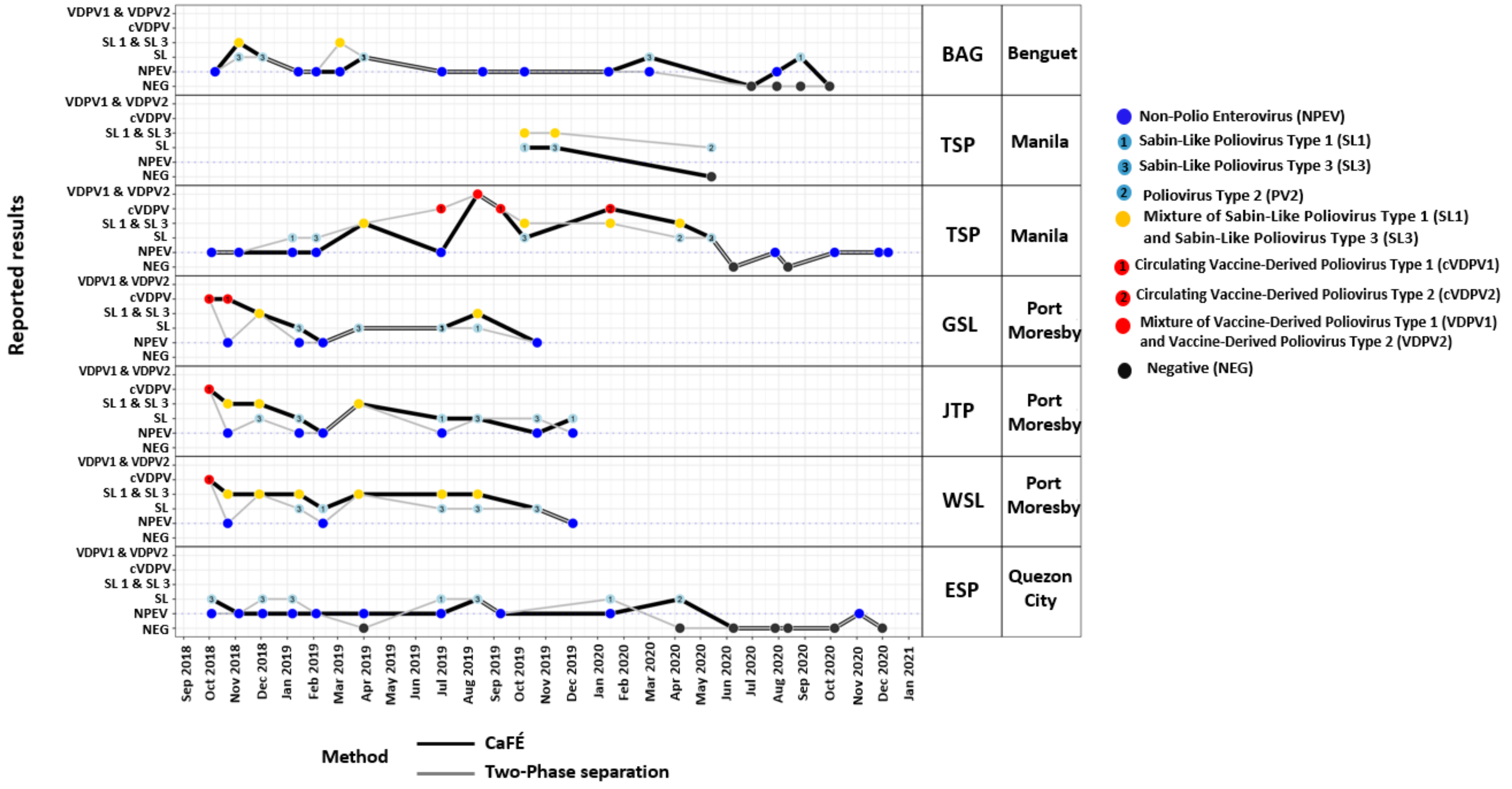


**Supplementary Figure 4.** Enteroviruses isolated through the Philippines (October 2018 - December 2020) and Papua New Guinea (October 2018 - December 2019) pilot testing for the CaFÉ method by month, year, sampling sites (the Philippines - Benguet: BAG = Baguio Sewage Treatment Plant, Quezon City: ESP = East Avenue Sewage Treatment Plant. Manila: ESP = East Avenue Sewage Treatment Plant, TSP = Tondo Sewage Pumping Plant. Papua New Guinea - Port Moresby: GSL = Gerehu Sewage Lagoon, JTP = Joyce Bay Treatment Plant, WSL = Waigani Sewage,), results, and the samples concentration method (two-phase separation and CaFÉ). ● NPEV = Non-Polio Enterovirus.

❶ SL1 = Sabin-Like Poliovirus Type 1. ❸ SL3 = Sabin-Like Poliovirus Type 3. ❷ SL2 = Poliovirus Type 2. ● SL1 & SL3 = Mixture of Sabin-Like Poliovirus Type 1 and Sabin-Like Poliovirus Type 3. ❶ cVDPV1 = Circulating Vaccine-Derived Poliovirus Type 1. ❷ cVDPV2 = Circulating Vaccine-Derived Poliovirus Type 2. ● VDPV1 & VDPV2 = Mixture of Vaccine-Derived Poliovirus Type 1 and Vaccine-Derived Poliovirus Type 2. ● NEG = Negative.
